# Generation and Observation of Long-Lasting and Self-Sustaining Marangoni Flow

**DOI:** 10.1021/acs.langmuir.3c00634

**Published:** 2023-05-25

**Authors:** Nikolaus Doppelhammer, Stefan Puttinger, Nick Pellens, Thomas Voglhuber-Brunnmaier, Karel Asselman, Bernhard Jakoby, Christine E. A. Kirschhock, Erwin K. Reichel

**Affiliations:** † Institute for Microelectronics and Microsensors, Johannes Kepler University Linz, Altenbergerstraße 69, 4040 Linz, Austria; ‡ Centre for Surface Chemistry and Catalysis: Characterization and Application Team, KU Leuven, 3001 Leuven, Belgium; § Department of Particulate Flow Modelling, Johannes Kepler University Linz, Altenbergerstraße 69, 4040 Linz, Austria

## Abstract

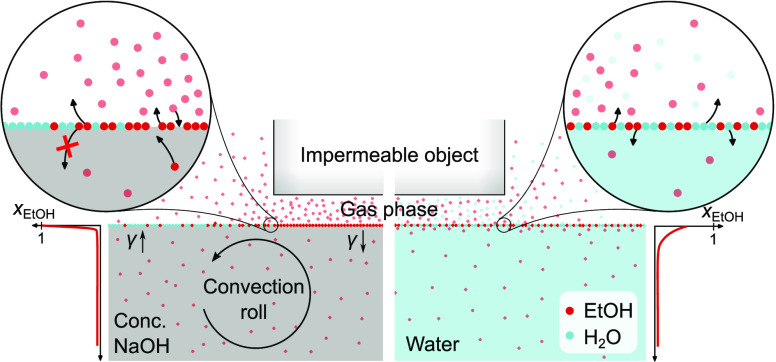

When solute molecules in a liquid evaporate at the surface, concentration gradients can lead to surface tension gradients and provoke fluid convection at the interface, a phenomenon commonly known as the Marangoni effect. Here, we demonstrate that minute quantities of ethanol in concentrated sodium hydroxide solution can induce pronounced and long-lasting Marangoni flow upon evaporation at room temperature. By employing particle image velocimetry and gravimetric analysis, we show that the mean interfacial speed of the evaporating solution sensitively increases with the evaporation rate for ethanol concentrations lower than 0.5 mol %. Placing impermeable objects near the liquid–gas interface enforces steady concentration gradients, thereby promoting the formation of stationary flows. This allows for contact-free control of the flow pattern as well as its modification by altering the objects shape. Analysis of bulk flows reveals that the energy of evaporation in the case of stationary flows is converted to kinetic fluid energy with high efficiency, but reducing the sodium hydroxide concentration drastically suppresses the observed effect to the point where flows become entirely absent. Investigating the properties of concentrated sodium hydroxide solution suggests that ethanol dissolution in the bulk is strongly limited. At the surface, however, the co-solvent is efficiently stored, enabling rapid adsorption or desorption of the alcohol depending on its concentration in the adjacent gas phase. This facilitates the generation of large surface tension gradients and, in combination with the perpetual replenishment of the surface ethanol concentration by bulk convection, to the generation of long-lasting, self-sustaining flows.

## Introduction

Surface tension gradients at liquid–liquid or liquid–gas interfaces induced by concentration gradients can lead to directed and collected transfer of molecules, commonly known as the solutal or concentration-driven Marangoni effect. Fundamental understanding of this effect is important as it plays a vital role in many biological processes^1^ and is crucial in the design and optimization of industrial applications such as the production of thin films and coatings,^2^ inkjet printing,^3^ and microfluidics.^4^

The classic experiment to demonstrate the solutal Marangoni effect is by adding a drop of dish soap on top of water, causing rapid spreading of the drop due to its lower surface tension compared to water. When concentration gradients are driven by the evaporation of solute molecules, more complex flow patterns can develop. Depending on various influences, among which the individual components volatility plays a crucial role, flows can manifest in different ways, involving toroidal convective flows in cylindrical pipes,^5^ vortical flows in sessile droplets,^6^ puncture and healing of liquid films,^7,8^ tears of wine,^9^ Marangoni-bursting^10^ and self-organization of nanoparticles.^11^

In evaporating systems, the gas and liquid phases often mutually affect each other. Complex mixing phenomena like fingering instabilities during the merging of miscible droplets^12^ or the dewetting behavior of thin and thick films^13^ crucially depend on the interplay of evaporation and (re-)condensation effects. Gas-phase-induced or -mediated Marangoni flows also facilitate “contactless” interaction between different liquid phases, enabling, for instance, Moses-like cleaving of droplets^14^ or control of droplet motility and position.^15^

A specifically interesting, yet unreported candidate for evaporation-driven, solutal Marangoni flow is concentrated sodium hydroxide solution mixed with minute quantities of ethanol, as discussed in this work. When nonuniform evaporation of such a mixture takes place, pronounced and long-lasting Marangoni flows develop. Although the resulting flows are chaotic when natural evaporation takes place, we will show that it is possible to control the flow behavior by enforcing steady concentration gradients in the gas phase. We explore the conversion between evaporation energy to kinetic fluid energy and further show that efficient conversion is only favored in solutions with high ionicity and low ethanol concentration. For this, we will provide an explanation that is supported by surface tension, conductivity, and viscosity measurements and is consistent with findings reported in the literature.

## Methods

### Main Experimental Setup

**Figure 1:**
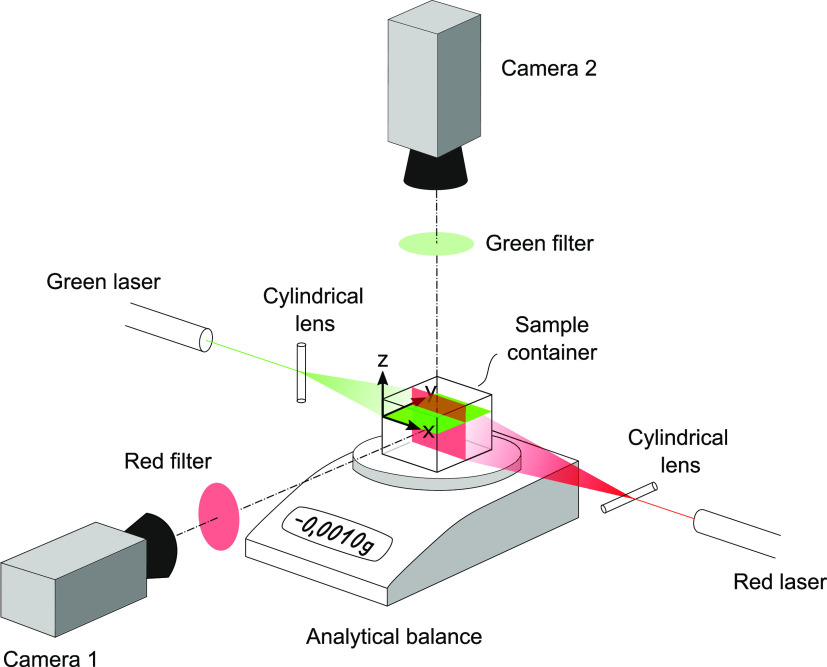
Combined dual-camera particle image velocimetry and gravimetry setup to study evaporation-induced Marangoni flows.

To study evaporation-driven Marangoni flows, a setup combining dual-camera particle image velocimetry (PIV)^16^ and gravimetric analysis, as illustrated in Figure 1, was employed. The sample solutions were filled into a custom container with transparent side walls and inner dimensions *W* × *B* × *H* = 27 mm × 27 mm × 24 mm. As a material, poly(methylmethacrylate) (PMMA) was selected due to its high translucency and excellent chemical resistance against alkaline media. To study flows, two laser sources from different angles were used: A green laser (neodymium-doped yttrium aluminum garnet (Nd:YAG, 532 nm, 0.5 W) to illuminated flows in a horizontal plane at the liquid surface and a second red laser (Flamenco, Cobolt, adjusted to 30 mW) to illuminate flows in a vertical plane bisecting the container. Two cameras, a Nikon Z6 and a Canon EOS 600D, were used to record fluid motion from the top and the side view, respectively. To avoid cross-talking of laser light, color filters were installed in front of the camera lenses. Movies from the top view were recorded at framerates between 60 and 120 fps, depending on the maximum expected flow speeds, with a resolution of 1920 × 1080 p. Movies from the side view were recorded at a frame rate of 50 fps with a resolution of 1280 × 720 p. This resulted in pixel densities of 31 and 23 px mm^–1^, respectively.

PIV processing of the image data was conducted in the software package Davis 10 (LaVision). Vector fields were calculated iteratively starting with an interrogation window size of 48 × 48 pixels down to a refined grid size of 24 × 24 pixels with 75% overlap. Surface- and time-averaged vector fields and the dissipation functions were computed in Python 3.0 using custom program code, which is available on request. In addition to PIV, the container was placed on a digital analytical balance with a resolution of 0.1 mg to monitor evaporative mass loss.

### Sample Preparation

A 17.5 mol % sodium hydroxide solution was prepared in 40 mL batches by mixing 17.25 g of sodium hydroxide pellets (reagent grade, Sigma-Aldrich) with 36.60 g of ultrapure water (Milli-Q). Then, ethanol was added to achieve the molar concentrations as specified in the main text. We did not account for the slight shift in sodium hydroxide concentration due to the addition of ethanol, but errors are negligible given the low concentration of alcohol in all experiments. The mass recipes of the studied samples are provided in the Supporting Information. To visualize flows for PIV, samples were seeded with hollow glass spheres (110P8, Potter Industries) at a concentration of 18 mg L^–1^, followed by rigorous stirring. For each experiment, the sample container was filled with 12.25 mL of liquid, corresponding to a sample height of 16.8 mm. All experiments and measurements were performed at a constant room temperature of 25 °C and 40% humidity.

### Pendant Drop Tensiometry

The surface tension of the test solutions in different gaseous environments was measured with an optical contact angle measurement device (OCA 25, Dataphysics). For the generation of pendant drops, the device’s automatic dosing system and a needle with a filling volume of 1 mL and a blunt tip (Sterican 0.8 mm × 22 mm, B.Braun) were used. Surface tension was calculated by fitting the Young–Laplace equation using the SCA 20 software from Dataphysics. The room-temperature density of the samples, required for surface tension measurements via this method, was determined with an EasyDens digital density meter from Anton Paar.

### Rheometry

Viscosity measurements, needed to calculate dissipated energy due to fluid motion, were performed on a Haake Mars 3 Rheometer (Thermo Fisher Scientific) with a C60/1° Ti L cone-plate cylinder geometry and active Peltier temperature control. Two avoid drying out of the samples, a sample hood in combination with a solvent trap ring (Thermo Fisher Scientific) filled with distilled water was used. Viscosity was measured at 10 logarithmically spaced shear rates in the range of 300 to 800 s^–1^. To check for repeatability, the shear rates were first increased, then held constant at 800 s^–1^ for 30 s, and decreased back to 300 s^–1^ again.

### Conductivity Measurements

Electric conductivity was measured using a custom setup employing the method of moving electrode electrochemical impedance spectroscopy (Supporting Information). For each measurement, 10 mL of sample solution was loaded into the preheated setup, corresponding to a sample height of 12.73 cm, followed by 30 min of phase and temperature equilibration. Monophasic samples were measured at electrode distances of *d*_e_ = 2, 2.25, 2.5,..., 7 cm. For biphasic samples, the measurement range was extended to *d*_e_ = 1, 1.25, 1.5,..., 8.5 cm. Only the impedance values where the moving electrode was entirely immersed in the bottom phase were selected for conductivity calculation. To exclude an influence of the moving electrode, e.g., by contamination of liquid that is dragged from one phase into the other, the conductivity of the lower phase was measured two times, one time in the direction of increasing electrode distances starting from the lowest position, and a second time by decreasing the electrode position starting from the most elevated position.

## Results

**Figure 2:**
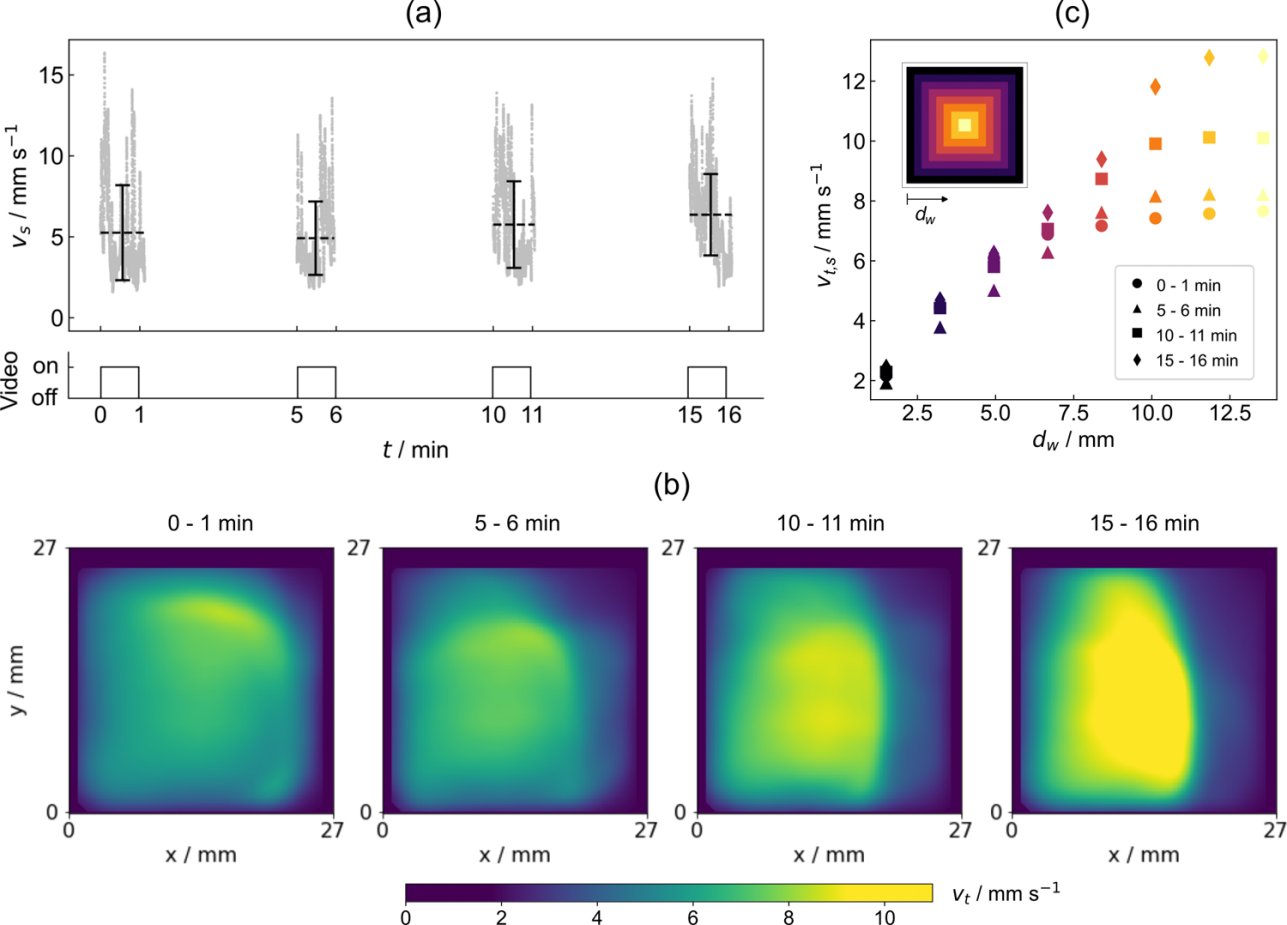
Analysis of interfacial flows in the generic case of SCS mixed with 0.5 mol % ethanol. (a) Surface-averaged flow speed as a function of time. Single gray dots refer to the average flow speed evaluated at time *t*. The dashed lines and error bars refer to the mean and standard deviation values, evaluated in one-minute intervals, respectively. (b) Time-averaged flow speeds at the surface, evaluated for the different time intervals. (c) Surface- and time-averaged flow speeds, evaluated in concentric regions at the surface.

At first, the generic case where 17.5 mol % sodium hydroxide solution, named showcase solution (SCS) hereinafter, was mixed with 0.5 mol % of ethanol is discussed. Once evaporation was permitted, in our case by removing a lid that covered the sample container, interfacial flows were triggered (Supporting Video 1). At the beginning (seconds 4 to 30 in the video), random popping up of flow sources at the interface was observed. Interfacial flow speeds in this initial phase reached maximum values of 30 mm s^–1^. Thereafter, flows changed to a steadier, swarmlike behavior. Evaluating the mean of surface-averaged flow speeds in periodic time intervals, represented by the dashed lines in Figure 2a, reveals that values stayed approximately constant over the entire observation time (16 min). Time-averaged flow speeds at the surface, as displayed in Figure 2b,c, reveal that speeds were initially rather uniformly distributed at the surface, whereas at a later stage, they became more localized. The latter presumably resulted from extensive swarmlike motion, where large convection rolls with high inertia formed in the bulk, leading to an abundance of flows in certain areas. The small rectangular shape of the container may have further amplified this effect. Figure 2b,c reveals that flows decayed to zero close to the walls, which indicates that the generation of surface tension gradients does not depend on a curved interface, as it is crucial in other evaporation-driven systems.^5,6,10,17−19^

**Figure 3:**
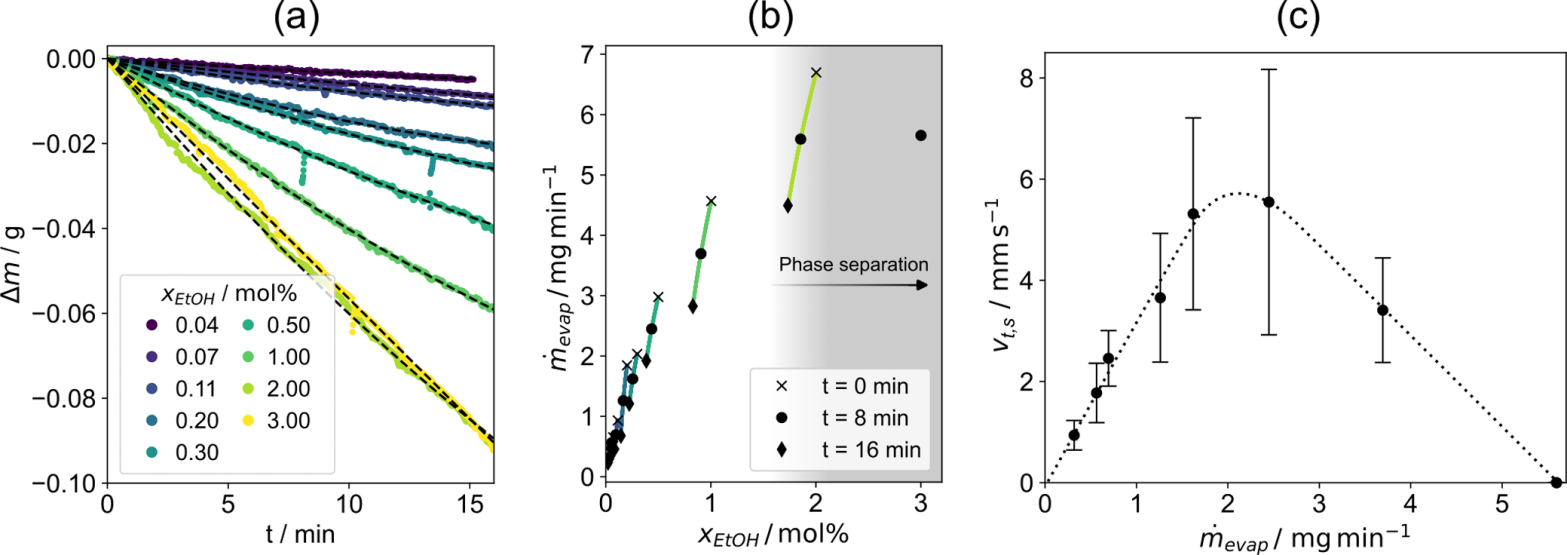
(a) Evaporative mass loss of SCS mixed with ethanol at various concentrations and respective curve fits (dashed curves) (b) Evaporation rate as a function of the ethanol concentration. (c) Mean surface speed as a function of the mean evaporation rate. *v*_t,s_ again denotes the time- and surface-averaged interfacial flow speed, this time evaluated for the entire surface and measurement period (16 min). Similar to Figure 2a, error bars represent the standard deviation of *v*_s_, corresponding to the mean intensity of speed fluctuations. The dotted line is a guide to the eye.

In a second experiment, the correlation between ethanol concentration and average interfacial flow speeds was investigated. Therefore, mixtures of SCS with varying amounts of ethanol, ranging from 0 to 3 mol %, were prepared. Figure 3a displays the mass loss over time for all tested samples. Samples with an ethanol concentration ≤2 mol % show a slightly nonlinear behavior in the form of a positive curvature, indicating a change in the sample composition. Assuming ethanol being the only evaporating component, as will be justified later, time-dependent functions of the evaporation rate and the molar ethanol fraction can be determined (Supporting Information). The evaporation rate as a function of the ethanol concentration, as illustrated in Figure 3b, shows that in the presence of ethanol at a low concentration, the evaporation rate *ṁ*_evap_ sensitively increased with the ethanol content, whereas at higher concentrations, a plateauing behavior was observed. Figure 3b also shows that, by comparing the evaporation rates between different samples and different points in time, the data does not collapse on a single curve. This indicates that the evaporation rate is not solely determined by the ethanol concentration, as samples that have already evaporated for a longer time display disproportionally lower evaporation rates than samples with a lower ethanol concentration at the beginning of the experiment. The reason for this may lie in evaporative cooling of the sample surface, decreasing the sample temperature over time and consequently leading to a reduction in the evaporation rate. Alternatively, there could be other effects related to sample aging that have yet to be understood. The 3 mol % sample shows a highly linear behavior in Figure 3a, i.e., a constant evaporation rate, which was confirmed for an extended measurement interval of 30 min. This sample, however, is already well within the region where macroscopic phase separation takes place, forming a top ethanol–water layer, and a dense sodium hydroxide solution with residual amounts of ethanol at the bottom. The correction in terms of a declining ethanol concentration over time was thus omitted for this sample.

Figure 3c shows the mean surface speed as a function of the mean evaporation rate. Note that *v*_t,s_, in this case, refers to the time- and surface averaged speed, evaluated for a single sample over the entire measurement period (16 min). Mean evaporation rates were determined by evaluating the slope of linear fit functions for the curves in Figure 3a. As visible, mean speeds and mean fluctuation intensities increased with the evaporation rate up to the sample with an initial ethanol concentration of 0.5 mol %. At higher concentrations, the flow speeds decreased again, despite still increasing evaporation rates. Around a concentration of 2 mol %, which is approximately the concentration where macroscopic phase separation sets in, self-sustaining flows at the interface became entirely absent.

**Figure 4:**
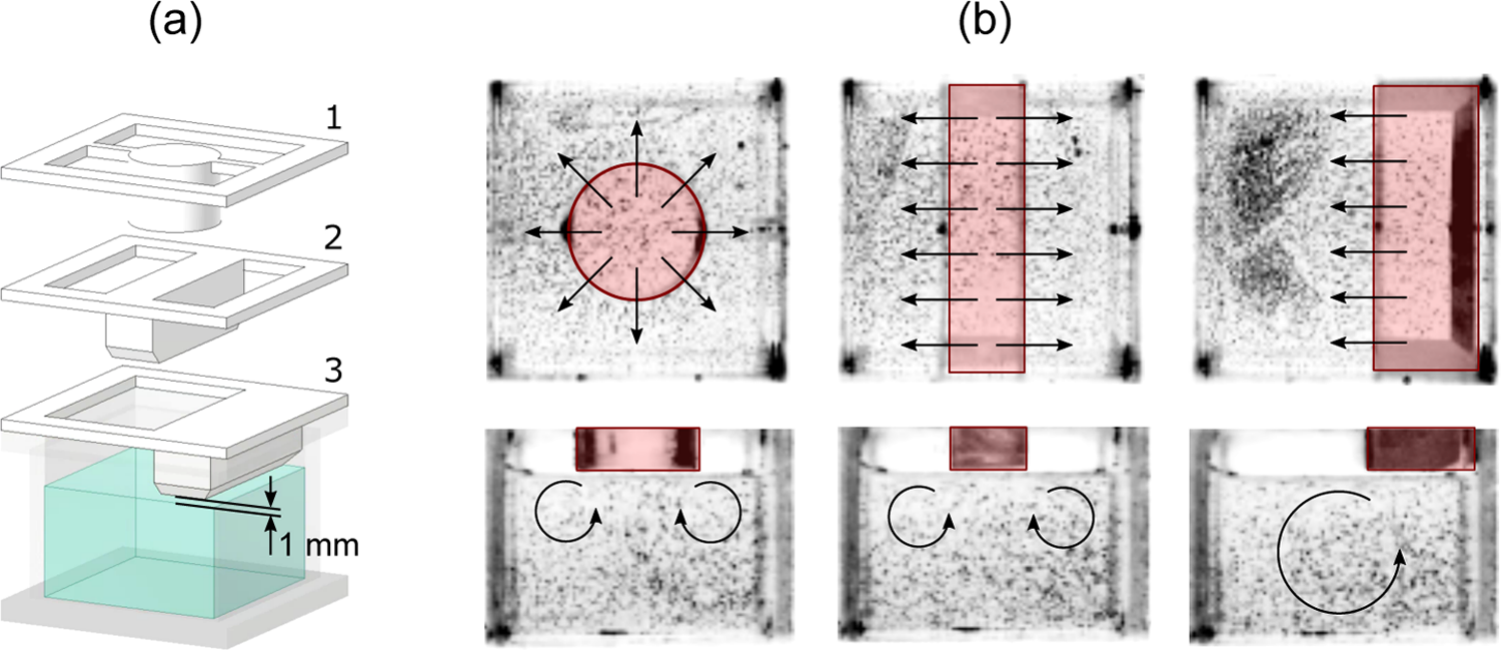
(a) Different geometries of impermeable objects. Objects 2 and 3 are chamfered on the side to avoid wetting in the presence of menisci at the side walls. (b) Snapshots of stationary flows created with the impermeable objects in (a). The sample in the displayed case is SCS mixed with 0.5 mol % ethanol. Red areas mark the locations where evaporation of ethanol molecules is locally hindered.

Our initial observations already suggest a strong correlation between surface tension gradients and evaporation rates. In the case of a freely evaporating liquid, mass flow across the liquid–gas interface is uncontrolled, thus leading to chaotic flows. Now we want to show that by controlling the ethanol concentration in the gas phase, it is possible to generate stationary flows with adjustable flow pattern. In contrast to organized transport through the liquid phase,^18,20^ evaporation-induced Marangoni flows offer a “contactless” way of controlling the flow behavior by placing impermeable objects close to the surface, thereby locally hindering the evaporation of ethanol. Consequently, ethanol is enriched at locations where an impermeable wall is close to the liquid surface, resulting in steady surface tension gradients and thus also in stationary Marangoni flows. The impermeable objects were laser-cut from translucent PMMA in three different shapes, as illustrated in Figure 4a, and placed at a distance of 1 mm away from the evaporating surface. Supporting Video 2 shows the flow patterns generated with each object. Snapshots of the patterns are displayed in Figure 4b, including marked areas to highlight the locations where evaporation is inhibited due to the presence of an impermeable wall.

As visible in all cases, flows with large, stationary convection rolls in the bulk liquid formed. The cylindrical object led to radial flow on the surface, whereas objects 2 and 3 led to parallel interfacial flows, as shown in Figure 4a. At the surface, flows in all cases were at right angles to each object’s boundary and directed to the free evaporating surface, implying a positive surface tension gradient toward the center of the container.^21^ This is reasonable because molecules in a liquid with high surface tension tend to pull more strongly on surrounding molecules than those in a liquid with lower surface tension, leading to the dragging of molecules away from areas of lower surface tension. The lowering of the surface tension underneath an impermeable object due to the enrichment of ethanol at the gas–liquid interface was confirmed by pendant drop experiments (Supporting Information). In the case of a hanging drop of SCS surrounded by air, a surface tension of γ_SCS,air_ = 90.28 mN m^–1^ was measured, whereas when the surrounding gas phase was enriched by evaporating ethanol, surface tension drastically reduced to γ_SCS,EtOH_ = 22.64 mN m^–1^. In comparison to water, the difference is less pronounced. Here, values of γ_H_2_O,air_ = 71.93 mN m^–1^ and γ_H_2_O,EtOH_ = 34.20 mN m^–1^ were measured.

In the case of stationary flows, the amount of evaporation energy (enthalpy of vaporization) converted into kinetic fluid energy can be estimated. Since fluid viscosity causes the dissipation of kinetic energy into heat, a fluid in motion loses speed unless external, propelling forces, in our case the interfacial shear stresses caused by the Marangoni effect, are present. Calculating dissipated energy requires the viscosity of the liquid and velocity information in 3D. The former was determined to μ = 12.64 mPa s with a rheometer for SCS mixed with 0.5 mol % of ethanol (Supporting Information). The latter normally cannot be evaluated from a 2D PIV experiment, but for quasi-two-dimensional flows, as those generated by the objects 2 and 3 in Figure 4a, where velocities only have components in *x*- and *z*-direction, 2D PIV data is sufficient (Supporting Information). Evaluating the dissipated energy over the entire sample volume gives values of *Ė*_kin,diss_ = 4.1 and 3.6 mJ s^–1^ for impermeable objects 2 and 3, respectively. Dividing these values by the evaporation rates yields the dissipated kinetic energy per unit mass of evaporated solvent  = 153 J g^−1^ and 134 J g^−1^. Under the assumptions of ethanol being the only evaporating component and the vapor pressure being identical to that of the neat substance, these results can be compared to the enthalpy of vaporization of ethanol *h*_e,EtOH_ = 846 J g^–1^,^22^ which can be seen as the maximum energy available for conversion into fluid motion, hence, an efficiency factor of the form
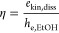
can be defined, giving values of 16.6% for flows generated with object 2 and 14.6% for those generated with object 3.

## Discussion

Efficient conversion of evaporation energy into fluid motion was exclusively observed at high sodium hydroxide concentration. In mixtures with lower ion concentration, i.e., higher water content but equimolar fractions of ethanol, interfacial flows were less sustained or entirely absent (Supporting Information). To explain the difference in behavior, the properties of water–ethanol mixtures in the presence and absence of sodium hydroxide should be discussed. It is known that concentrated alkali hydroxide solutions tend to phase-separate upon addition of alcohol due to salting-out of the organic phase, commonly observed in concentrated electrolyte systems.^23,24^ Phase separation leads to the formation of two liquid phases, an alcohol–water phase on the top, and a dehydrated sodium hydroxide phase at the bottom. The latter is capable of storing trace amounts of ethanol before phase separation is triggered, as revealed by conductivity measurements (Supporting Information). In the case of SCS, the maximum ethanol concentration to be stored in the bulk liquid was found to be around 2 mol % before the mixture started to phase-separate. At the interface, a different situation arises. The surface tension of SCS is lowered approximately to the value of neat ethanol (γ_neat EtOH_ = 21.91 mN m^–1^^22^) when the surrounding gas phase is enriched with ethanol at ambient pressure (Supporting Information). This implies that the surface of SCS is entirely covered by ethanol molecules in this environment. Concentrated sodium hydroxide solution therefore acts as a good adsorbent, where ethanol can be efficiently stored at the interface, but as a bad absorbent, tolerating only a low concentration of the alcohol in the bulk liquid. According to the Gibbs adsorption theory,^25^ a solute that increases a liquid’s surface tension implies a negative surface excess concentration, i.e., a lower concentration of the compound at the interface than in the bulk. This is the case for sodium hydroxide solution, where addition of NaOH leads to an increase in surface tension (in the case of air being the surrounding gas phase). Molecular dynamics simulations have confirmed this behavior,^26^ further revealing that the first molecular layer at the surface mainly consists of water, whereas the concentration of ions, both sodium and hydroxide, rapidly increases at increasing distances away from the surface. Moreover, the researchers found that the number of free OH bonds at the surface, even at high NaOH concentration, is approximately identical to that of neat water, explaining the high affinity for adsorption/desorption of ethanol, whose interaction with water is mainly via H-bonding. These findings in combination with surface tension measurements suggest that the interface of concentrated sodium hydroxide solution constitutes only a thin (mono)layer entirely composed of ethanol when the adjacent gas phase is rich in ethanol. When evaporation takes place, the ethanol readily desorbs from the liquid surface, whereas water, being highly polar and therefore much more strongly bound to the dissolved ions and other water molecules, evaporates to a much lesser extent. As a result, large and fast surface tension gradients are created, triggering pronounced interfacial flows. Marangoni flows imply the creation of a new interface “drawn” out of the bulk liquid, best understood by looking at the stationary flow patterns generated by the impermeable objects in Figure 4b. This leads to convective transport of ethanol molecules from the bulk to the surface, thereby replenishing the ethanol inventory at locations where flows are directed toward the surface. Ethanol transport due to diffusion, which would lead to less pronounced surface tension gradients because ethanol is also replenished in areas where the bulk flow underneath is not necessarily directed toward the evaporating surface, presumably only has a weak influence, given the high viscosity of concentrated sodium hydroxide solution, which drastically reduces molecular diffusivity. Despite the small quantities of ethanol in our systems, evaporation only slowly depletes the ethanol concentration in the bulk, as shown in Figure 3b, explaining the self-sustaining and long-lasting flow behavior.

In water–ethanol systems, or mixtures with low ion concentration, the situation is different. Since ethanol diffusion into the bulk is not hindered by the presence of ions, this results in a less steep concentration profile of the co-solvent in the liquid phase normal to the surface. The former can again be deduced from pendant drop measurements, where water adjacent to ethanol-saturated air was found to have an 11.56 mN m^–1^ higher surface tension than SCS in the same environment (Supporting Information). Molecular dynamics simulations of ethanol–water mixtures confirmed, that, albeit the surface concentration increases nonlinearly with the ethanol bulk concentration, the ethanol concentration at the surface is much lower compared to SCS.^27^ This implies that, when evaporation takes place, ethanol and water molecules are transferred to the gas phase. In fact, at a low ethanol concentration, mainly water evaporates in these systems.^17^ The composition of the liquid at the surface thereby changes, if any, only to a little extent, and surface tension gradients are not high enough to trigger self-sustaining Marangoni flows. Due to the much lower viscosity of water compared to SCS, local concentration imbalance in the liquid phase may additionally be counteracted by diffusive processes, which are known to stabilize evaporating systems against Marangoni flow.^5,28,29^ The differences in properties and behavior of the two extreme cases, water and SCS mixed with small but equimolar amounts of ethanol, are explained graphically in the ToC.

## Conclusions

In this work, we have shown that evaporation of concentrated sodium hydroxide solution mixed with ethanol at a very low concentration triggers long-lasting and self-sustaining Marangoni flow. The chaotic flow behavior can be controlled by placing impermeable objects close to the liquid surface which enforce stationary concentration gradients in the gas phase, thereby generating flows with adjustable pattern. Our observations can be explained by inspecting the specific properties of concentrated sodium hydroxide solution, where only a small amount of ethanol is tolerated in the bulk liquid, whereas at the surface, the co-solvent is stored in a thin (mono)layer, allowing for a high concentration of the alcohol and its quick ad- and desorption, depending on the concentration in the surrounding gas phase. In combination with the drastically lower surface tension of ethanol than sodium hydroxide, this facilitates the generation of large surface tension gradients. Due to the perpetual replenishment of ethanol at the surface through convective flow, self-sustaining and long-lasting flows are generated.

## Supporting Information Available

Pendant drop experiments; calculation of dissipated viscous energy; viscosity measurements; conductivity measurements; influence of reduced salt concentration; calculation of the molar ethanol fraction and evaporation rate; assessment of thermocapillary effects; and other supporting data (PDF)
Interfacial flows at the free evaporating surface (Video 1) (MP4)
Effect of impermeable objects in the gas phase on the developing flow pattern (Video 2) (MP4)
