# Supplementary material for: Generation and Observation of Long-Lasting and Self-Sustaining Marangoni Flow
Source: Langmuir. 2023 May 25;39(22):7804–10. doi: 10.1021/acs.langmuir.3c00634 (PMC10249401; doi:10.1021/acs.langmuir.3c00634)
Supplement: Supplementary file 1 — la3c00634_si_001.pdf [file la3c00634_si_001.pdf]

Supporting Information:

Generation and Observation of Long-lasting and Self-sustaining Marangoni Flow

Nikolaus Doppelhammer[1,2,], Stefan Puttinger[3], Nick Pellens[2], Thomas Voglhuber-Brunnmaier[1], Karel Asselman[2], Bernhard Jakoby[1], Christine E.A. Kirschhock[2], Erwin K. Reichel[1].*

1: Institute for Microelectronics and Microsensors, Johannes Kepler University Linz, Altenbergerstraße 69, 4040 Linz, Austria.

2: Centre for Surface Chemistry and Catalysis: Characterization and Application Team, KU Leuven, 3001 Leuven, Belgium

3: Department for Particulate Flow Modelling, Johannes Kepler University Linz, Altenbergerstraße 69, 4040 Linz, Austria.

*: Corresponding author

Contents

Pendant drop experiments ... 2
Calculation of dissipated viscous energy ... 3
Viscosity measurements ... 4
Conductivity measurements ... 6
Influence of reduced salt concentration ... 8
Calculation of molar ethanol fraction and evaporation rate ... 9
Assessment of thermocapillary effects ... 10
Other supporting data ... 11
References ... 11

Pendant drop experiments

To simulate the influence of ethanol in the gas phase, a petri dish filled with ethanol was placed directly under a freshly generated drop of sample liquid, as illustrated in Figure S1. Due to the close distance of the drop to the evaporating surface, fast equilibrium between the gas and liquid phase was achieved. For each experiment, the mean of six measurement values was taken. All measurement values are provided in Table S1.

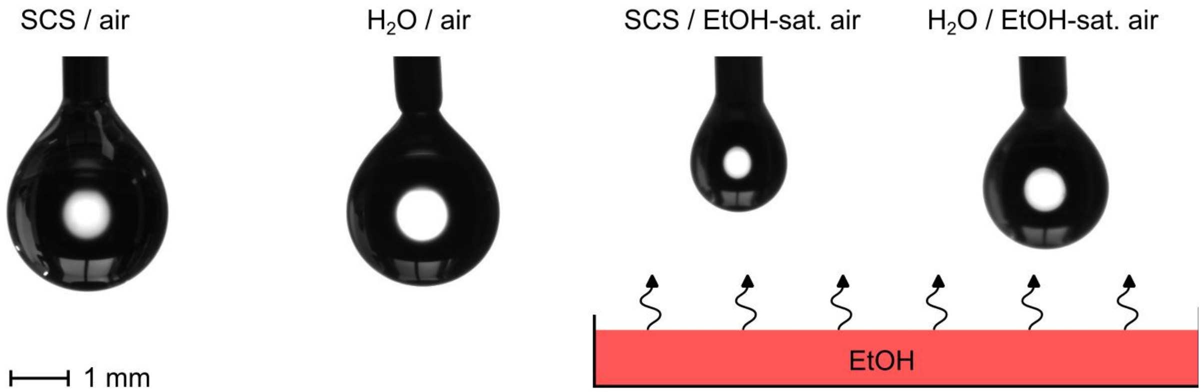

Figure S1. Selected snapshots of pendant drops for surface tension measurements of SCS and water in air and ethanol-enriched air.

γ / mN m^{-1}			
SCS / air	H2O / air	SCS / EtOH-sat. air	H2O / EtOH-sat. air
89.81	70.84	22.68	28.20
91.24	70.71	24.74	36.08
90.93	71.68	19.43	37.24
92.27	72.19	22.54	35.18
90.89	72.98	23.53	42.19
86.52	73.19	22.90	26.31
Mean			
90.28	71.93	22.64	34.20

Table S1. Surface tension values.

Calculation of dissipated viscous energy

The amount of dissipated kinetic energy per unit time can be calculated from the velocity field by[1]

$$\dot{E}_{kin,diss} = -\int \phi \, dV, \tag{1}$$

where ϕ is the dissipation function for an incompressible fluid with the general form

$$\phi = \mu \left[2\left(\frac{\partial u}{\partial x}\right)^2 + 2\left(\frac{\partial v}{\partial y}\right)^2 + 2\left(\frac{\partial w}{\partial z}\right)^2 + \left(\frac{\partial w}{\partial y} + \frac{\partial v}{\partial z}\right)^2 + \left(\frac{\partial u}{\partial z} + \frac{\partial w}{\partial x}\right)^2 + \left(\frac{\partial v}{\partial x} + \frac{\partial u}{\partial y}\right)^2 \right].$$

Here, u, v and w denote the velocity components in x, y and z-direction, respectively. The bulk viscosity of SCS (17.5 mol% NaOH solution) mixed with 0.5 mol% of ethanol $\mu = 12.64\ \mathrm{mPa\ s}$ was determined by rheometry. For flows with velocity components only in the x- and z-direction, the dissipation function simplifies to

$$\phi_{xz} = \mu \left[2\left(\frac{\partial u}{\partial x}\right)^2 + 2\left(\frac{\partial w}{\partial z}\right)^2 + \left(\frac{\partial u}{\partial z} + \frac{\partial w}{\partial x}\right)^2 \right]. \tag{2}$$

To calculate the dissipated kinetic energy from PIV velocity fields, Equations 1 and 2 were implemented in discretized form in MATLAB (r2020a, The MathWorks Inc.). The code is available on request. The dissipation functions for the flows generated by vapor trap geometries 2 and 3 for SCS mixed with 0.5 mol% of ethanol are provided in Figure S2.

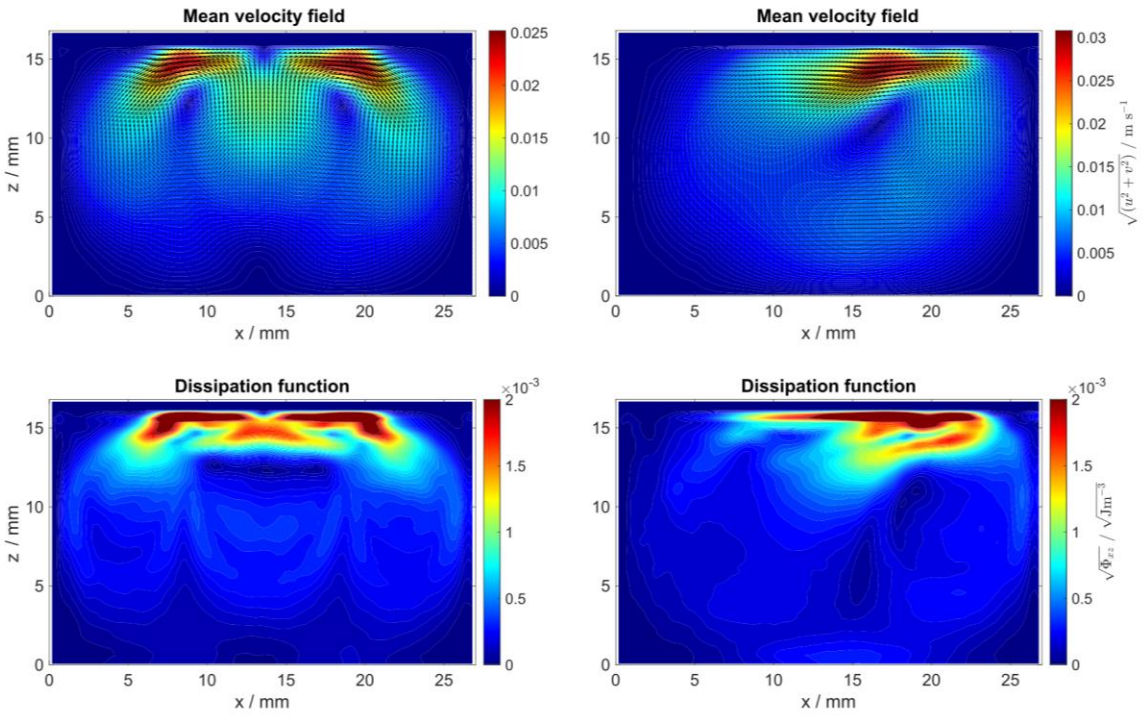

Figure S2. Mean velocity fields and dissipation functions of stationary flows generated with vapour trap geometries 2 (left) and 3 (right). The liquid sample in this case is SCS mixed with 0.5 mol% of ethanol. Dissipation function values were truncated at $2\,x\,10^{-3}\sqrt{\mathrm{J\,m^{-3}}}$ to enhance visibility.

Viscosity measurements

To determine the influence of ethanol on the bulk viscosity of SCS, three samples with ethanol concentrations of 0, 0.5 and 1 mol% were tested. As visible in Figure S3, all samples showed approximately Newtonian behavior, i.e., constant viscosity as a function of the shear rate. This allows determination of viscosity simply via averaging over datapoints, yielding the values provided in Table S2. For the sample without ethanol, the value is in good agreement with data reported in the literature[2].

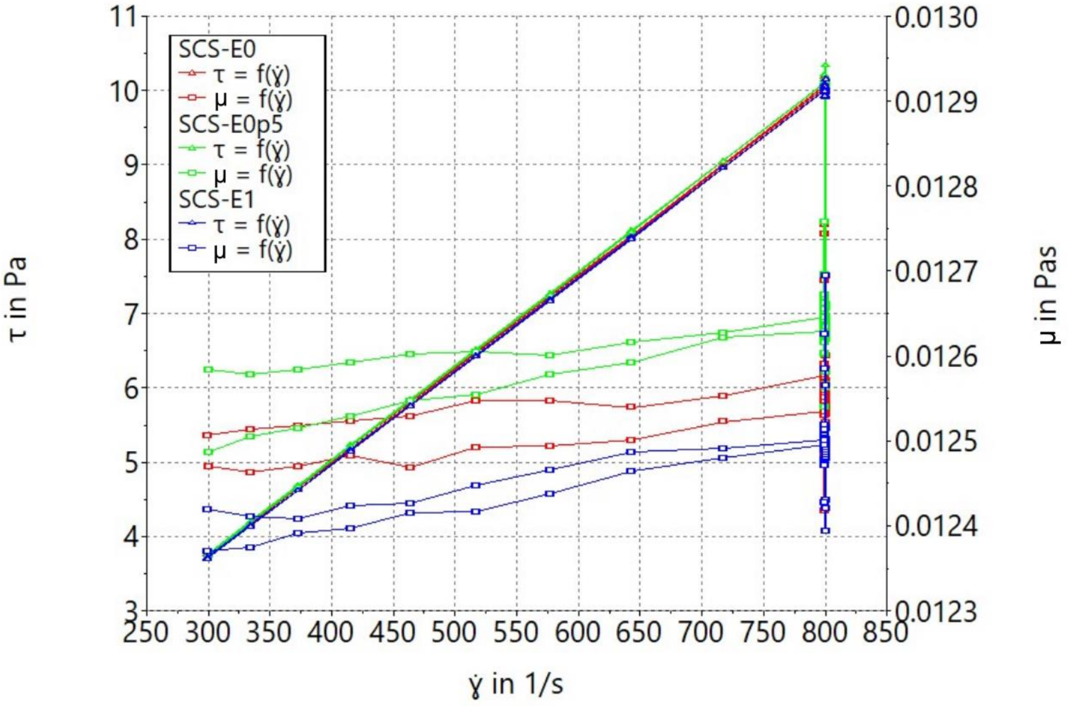

Figure S3. Shear stress τ and viscosity μ of SCS mixed with 0 (red), 0.5 (green) and 1 mol% (blue) of ethanol as functions of the shear rate $\dot{\gamma}$

Sample	μ / mPa s
SCS + 0.0 mol% EtOH	12.56
SCS + 0.5 mol% EtOH	12.64
SCS + 1.0 mol% EtOH	12.5

Table S2. Mean viscosity values of SCS with varying amounts of ethanol.

Conductivity measurements

To validate that concentrated sodium hydroxide solution can store small amounts of ethanol, the electrolytic conductivity was measured using a custom setup employing the method of moving electrode electrochemical impedance spectroscopy (MEEIS). In contrast to conductivity sensors with static electrodes, MEEIS uses two parallel, oppositely arranged electrodes, with one of them being mounted on a motorized linear stage. This enables precise adjustment of the electrode distance and allows determination of the conductivity by a modified version of Pouilett's law,

$$\sigma = \frac{1}{a\,A}, \tag{3}$$

where a is the slope of the impedance as a function of the electrode distance and A the (homogenous) cross section of the sample. Due to capacitive effects at the electrode-liquid interface, the measured impedance is frequency dependent and selection of the right value is crucial to obtain accurate results. Therefore, impedance spectra are recorded in a large frequency range (10 Hz to 5 MHz), and the measurement frequency is chosen based on a minimal-phase criterion[3]. Since conductivity is not determined from the absolute impedance value but the impedance increment as a function of the electrode distance, (quasi-)constant contributions to the impedance are eliminated, enabling accurate conductivity measurements of chemically aggressive, highly conducting and heterogenous media, as demonstrated in previous studies of our research groups[4–6]. Furthermore, this method allows locally probing the sample properties, enabling the analysis of individual phases, e.g., of a phase-separating sample.

When ethanol was added in small amounts to SCS (17.5 mol% NaOH solution), no visible phase separation was observed but conductivity drastically decreased, as shown in Figure S4. At a concentration of approximately 2 mol%, conductivity of the lower phase started to plateau, which coincides with the point where macroscopic phase separation was detected. The initial decrease in conductivity is presumably due to an increasing affinity for pairing between sodium and hydroxide ions in the presence of ethanol. Ion pairing in concentrated electrolyte solutions reduces the electrolytic conductivity, as the ions no longer act as free charges but rather as dipoles, therefore decreasing the ability of the ions to conduct electric current. Ion pairing is facilitated in solvents with a low dielectric constant[7]. Given the much lower dielectric constant of ethanol compared to water ($\varepsilon_{\mathrm{EtOH}} = 25.3, \varepsilon_{\mathrm{H_2O}} = 80.1$ [8]), conductivity decreases when ethanol is present in the bulk.

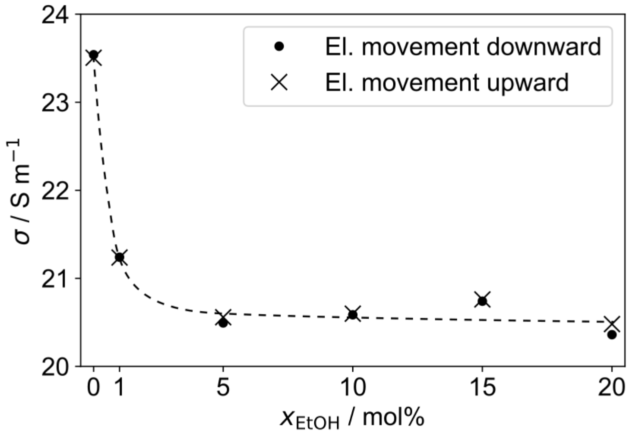

Figure S4. Bulk conductivity of SCS mixed with varying amounts of ethanol. Around an ethanol concentration of 2 mol%, phase separation is triggered, splitting the liquid into a dense concentrated sodium hydroxide bottom phase and top ethanol-water phase. Only the conductivity of the bottom phase is shown. The dashed line is a guide to the eye.

Influence of reduced salt concentration

To investigate the influence of salt concentration on interfacial flow speeds, a sample with reduced (10 mol% NaOH) and a sample without NaOH, each mixed with 0.5 mol% of ethanol, were investigated. At this ethanol concentration, flow speeds reached a maximum for the most concentrated sample (SCS + 17.5 mol% NaOH , cf. Figure 2(a)), whereas flow speeds for the sample with 10 mol% NaOH were already significantly lower and less sustained, as shown in Figure S5. When no salt was added (0 mol% NaOH), flows were entirely absent, although a similar mass loss due to evaporation was measured for all three samples (Figure S6).

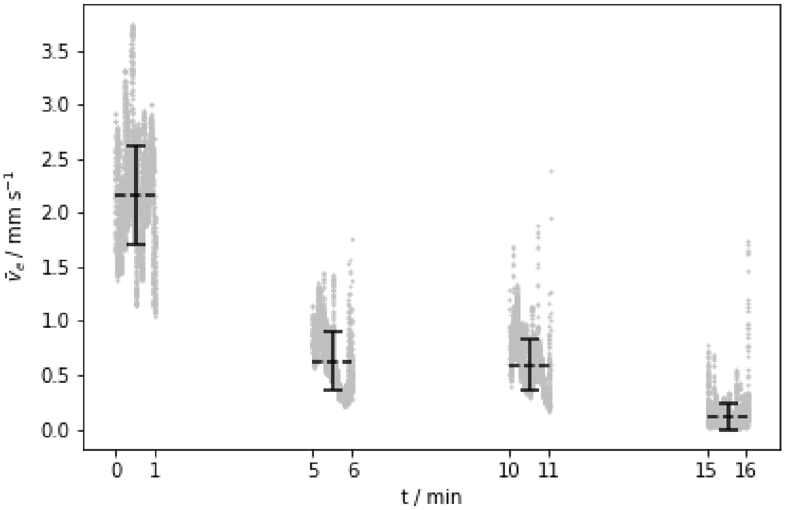

Figure S5. Surface-averaged velocities for a sample with reduced salt concentration ($x_{\mathrm{NaOH}} = 10$ mol%, $x_{\mathrm{EtOH}} = 0.5$ mol%), resulting in considerably lower and less sustained flow velocities than in the case of SCS. For water mixed with 0.5 mol% of ethanol, flows were entirely absent.

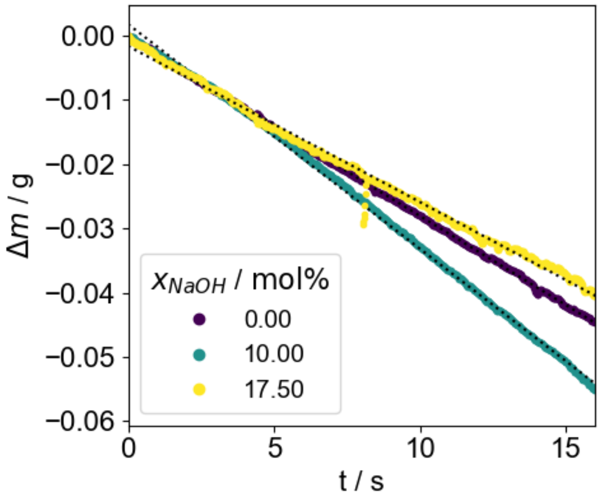

Figure S6. Mass loss due to evaporation for three samples with different NaOH concentration but equimolar ethanol concentration of 0.5 mol%.

Calculation of molar ethanol fraction and evaporation rate

First, each measurement curve was fitted with an appropriate function (dashed lines in Figure 3(a)). For concentrations ≤ 2 mol%, a quadratic function of the form $y = ax + bx^2$ was used to account for the slightly positive curvature, whereas for the 3 mol% samples, a linear function of the form $y = ax$ was used. Assuming ethanol being the only evaporating component, the ethanol mass in the sample as a function over time is given by

$$m_{EtOH}(t) = m_{EtOH}(t_0) - \Delta m(t), \tag{4}$$

where $m_{EtOH}(t_0)$ is the mass of ethanol in the sample before evaporation takes place, and $\Delta m(t)$ is the mass loss over time determined from the line fits. The molar ethanol fraction as a function of time is determined by

$$x_{EtOH}(t) = \frac{\frac{m_{EtOH}(t)}{M_{EtOH}}}{\frac{m_S - m_{EtOH}(t)}{M_{NaOH}} w_{NaOH} + \frac{m_S - m_{EtOH}(t)}{M_{H_2O}}(1 - w_{NaOH}) + \frac{m_{EtOH}(t)}{M_{EtOH}}} \tag{5}$$

where M_{EtOH}, M_{NaOH} and M_{H_2O} are the molar mass of ethanol, sodium hydroxide and water, respectively, $w_{NaOH} = 0.32$ is the weight fraction of NaOH in SCS, and m_s = 16.42 g is the initial sample mass, calculated as the product of the initial sample volume (12.25 ml) times the sample density ($\rho_{SCS} = 1.34$ g cm^{-1}).

Evaporation rates as a function of time were determined by differentiating the line fits over time using the Python's *numpy* package.

Assessment of thermocapillary effects

Evaporation of ethanol is accompanied by cooling of the liquid surface. Since evaporation is non-uniform, temperature gradients along the interface can be expected. It can be argued that the influence of temperature-induced Marangoni flows is low due to the high interfacial velocities observed in our experiments, leading to rapid compensation of local temperature imbalance by fluid mass transport. Furthermore, to experimentally investigate the existence of temperature-induced Marangoni flows, we conducted experiments using both a cold (0°C) and hot (330°C) solder iron, placing their tips approximately 1 mm close to the surface of SCS. In both cases, no visually noticeable interfacial flows were detected. Therefore, considering that temperature gradients resulting from evaporation are in the range of a few degrees Celsius, a potential influence of temperature-induced Marangoni flows can be disregarded.

Other supporting data

x_{EtOH} / mol%	m_{NaOH} / g	m_{H_2O} / g	m_{EtOH} / g
0.04	17.25	36.60	0.04
0.07	17.25	36.60	0.08
0.11	17.25	36.60	0.13
0.20	17.25	36.60	0.23
0.30	17.25	36.60	0.34
0.50	17.25	36.60	0.57
1.00	17.25	36.60	1.15
2.00	17.25	36.60	2.32
3.00	17.25	36.60	3.51

Table S3. Mass recipes for 17.5 mol% NaOH solutions with varying ethanol concentration

x_{EtOH} / mol%	m_{NaOH} / g	m_{H_2O} / g	m_{EtOH} / g
0.50	12.72	26.86	0.42
0.50	7.86	31.69	0.45
0.50	0.00	39.49	0.00

Table S4. Mass recipes for experiments with varying water/salt content

References

(1) Lamb H. *Hydrodynamics,* 6th ed.; Cambridge Univ. Press: Cambridge, 1995.

(2) Sipos, P. M., Hefter, G., May, P. M. Viscosities and Densities of Highly Concentrated Aqueous MOH Solutions (M + = Na + K + Li + Cs + (CH 3) 4 N +) at 25.0 °C. *J. Chem. Eng. Data* **2000**, *45*, 613–617.

(3) Doppelhammer, N., Pellens, N., Martens, J., Kirschhock, C. E. A., Jakoby, B., Reichel, E. K. Moving Electrode Impedance Spectroscopy for Accurate Conductivity Measurements of Corrosive Ionic Media. *ACS Sens* **2020**, *5*, 3392–3397.

(4) Doppelhammer, N., Pellens, N., Jakoby, B., Kirschhock, C. E., Reichel, E. K. Monitoring Zeolite Formation with Moving Electrode Conductometry. *2021 IEEE Sensors*; IEEE, 2021.

(5) Pellens, N., Doppelhammer, N., Asselman, K., Thijs, B., Jakoby, B., Reichel, E. K., Taulelle, F., Martens, J., Breynaert, E., Kirschhock, C. E. A. A zeolite crystallisation model confirmed by in situ observation. *Faraday Discuss.* **2022**, *235*, 162–182.

(6) Doppelhammer, N., Pellens, N., Kirschhock, C. E. A., Jakoby, B., Reichel, E. K. Using Moving Electrode Impedance Spectroscopy to Monitor Particle Sedimentation. *IEEE Sensors J.* **2021**, *21*, 9636–9641.

(7) Marcus, Y., Hefter, G. Ion pairing. *Chem. Rev.* **2006**, *106*, 4585–4621.

(8) Haynes W. M., Lide D. R., Bruno T. J. *CRC Handbook of Chemistry and Physics*; CRC Press, 2016.
